# Differences in breast carcinoma immunohistochemical subtypes between immigrant Arab and European women

**DOI:** 10.1186/1746-1596-9-26

**Published:** 2014-02-04

**Authors:** Fanny Preat, Philippe Simon, Jean-Christophe Noel

**Affiliations:** 1 Department of Senology, Erasme University Hospital-ULB, Route de Lennik 808, B-1070 Bruxelles, Belgium

**Keywords:** Arab, European, Breast carcinoma, Molecular classification, Luminal A, Luminal B, Immunohistochemistry

## Abstract

**Background:**

There is a dearth of information on the clinicopathological differences, including the molecular subtypes, of breast carcinomas from immigrant Arab women in Europe. Therefore, the aim of our study was to examine and compare these features in immigrant Arab/Moroccan patients with those of European women.

**Methods:**

Included in this study were 441 cases of breast cancer: 91 Arab/Moroccan women and 350 European women. Age, size, grade, node involvement, and immunohistochemical profile (classification into the following subtypes: luminal A, luminal B, HER2 +/ER -, and triple negative) were analyzed.

**Results:**

The average age of breast cancer presentation in Arab women is almost a decade earlier than in European women (49 versus 60 years old; p = 0.00001). Arab patients also had a higher average tumor size (25 mm versus 19 mm; p =0,008) and more grade 3 and less grade 1 tumors (p = 0.02). It should be noted, however, that this variability in the size and grade do not appear statistically significant when compared in Arab and European patients under 50 years old. In contrast, independent of age, the immunohistochemical subtypes were different between the two populations, with a greater number of luminal B subtype and fewer luminal A subtype (p <0.02) in Arab patients.

**Conclusions:**

Arab patients with breast carcinoma have different clinicopathological features from European patients, mainly the age of cancer presentation. Their immunohistochemical profile is also different, with more luminal B and less luminal A subtypes, suggesting that there are not only clinicopathological differences but also disparities in the expression profiling in these women.

**Virtual slide:**

The virtual slides for this article can be found here: http://www.diagnosticpathology.diagnomx.eu/vs/2104813621113288.

## Background

In Belgium, breast cancer accounts for almost 13% of all cancers and each year 9,400 new cases are observed. Its incidence is estimated at 146/100,000 [1]. The origin of this cancer is likely multifactorial and many factors have been implicated, including the woman’s reproductive lifestyle, endogenous hormones, exogenous hormones, adiposity, physical activity, nutrition, alcohol consumption, smoking, environmental toxins, and genetic susceptibility in inherited syndromes [2]. Breast cancer rates also differ by race and ethnicity [3]. It has been suggested that breast cancer in Arab populations has specific morphological and molecular characteristics, including poorly differentiated pathological features and increased HER2 overexpression [4]. Naturally, Arab populations are not homogeneous and have genetic diversity [5]. However, in a recent publication El Fatemi et al. demonstrated that in a Moroccan Arab population, the luminal B subtype was the most prevalent [6]. These studies, however, were performed on the indigenous population, and there are no data on the Moroccan Arab immigrant women in Western Europe. Since the Moroccan immigrant population is the most common at our institution, the aim of this study was to define the morphological and immunohistochemical (surrogates to molecular classes) characteristics of breast carcinomas in these immigrant Moroccan Arab patients and compare them with the European native population.

## Materials and methods

Breast carcinoma specimens in formalin-fixed, paraffin-embedded tissue blocks from 441 female patients diagnosed with invasive carcinoma from january 2008 to december 2012 were retrieved from the archives of the Department of Pathology, Erasme University Hospital. This study was approved by the local ethics committee (Erasme University Hospital, reference number: 2013/027) and included 350 European white women and 91 Moroccan Arab patients. The pathological stage and histological grade were defined according to the criteria of the World Health Organization 2012 [7]. The estrogen receptor (ER), progesterone receptor (PR), Ki-67 labeling index, and HER2 expression were evaluated at the time of the original diagnosis by immunohistochemistry, as previously described [8-10]. A clinically positive test for the receptors is defined as nuclear staining in ≥1% of the tumor cells [8]. HER2 immunoreactivity was performed using the Oracle HER2 test (clone CB11; Leica Microsystems GmbH, Wetzlar, Germany) according to the manufacturer’s instructions, as previously described [10]. The scoring was assessed with the recommendations of the American Society of Clinical Oncology [11]. All of the HER2 scores of 2+ and 3+ were analyzed using the fluorescent *in situ* hybridization (FISH) PathVysion HER2 DNA test (Abbott laboratories, Abbott Park, USA) according to the manufacturer’s instructions. Signal ratios (HER2/CEP17) of ≥2 were classified as amplified. In this study, only 2+ and 3+ tumors with a HER2 FISH amplification were considered as a positive result. A subtype immunohistochemical classification (surrogates to molecular classes) was adopted to characterize the tumors, using the following criteria: Luminal A, when either one or both of the ER and PR were present, HER2 was negative and Ki-67 <14%; Luminal B, when ER and/or PR were present and either Ki-67 ≥14% or HER2 was positive; HER2 positive, when ER and PR were absent and HER2 was positive irrespective of the Ki-67; and Triple negative, when ER and PR were absent and HER2 was negative. The correlation analysis was conducted using the χ^2^ test and Fisher’s exact probably test. For the comparison of the means, the student’s t-test was used. Results were considered significant when p <0.05.

## Results

**Figure 1:**
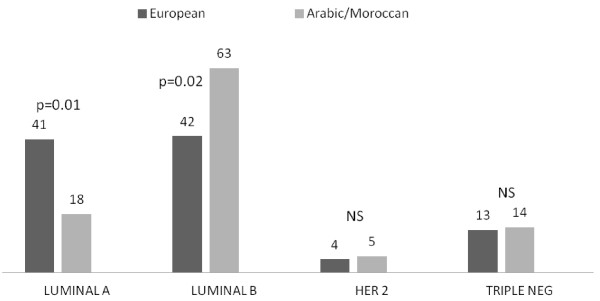
Immunohistochemical profile surrogates of the molecular classes in premenopausal women (in percentage).

The clinicopathological characteristics of the 441 patients are summarized in the Table 1. Arab/Moroccan patients were characterized by an average age of presentation for breast cancer almost a decade earlier than in European individuals (49 versus 60 years old; p = 0.00001). In addition, the size of the tumor in these patients was larger (25 mm versus 19 mm; p = 0.008), the incidence of grade 3 tumors more frequent (p = 0.01), the grade 1 less frequent (p = 0.02), the percentage of PR positive lesions was less (p = 0.02), the mean Ki-67 index was higher (27% versus 20%; p = 0.003), the Luminal A tumor was less frequent (p = 0.001), and the Luminal B was more frequent (p = 0.02). To ensure these results were not biased by the age at the time of the presentation of cancer, we only extracted premenopausal patients from this series. This enabled us able to compare groups with an almost identical age (43 versus 42 years old). Interestingly, despite these adjustments, the Arab/Moroccan patients continued to display less Luminal A tumors (p = 0.01) and more Luminal B tumors (p = 0.02) (Table 2) (Figure 1).

**Table 1:** Clinicopathological characteristics of the breast cancer patients (all ages combined)

	**European**	**Arabic/Moroccan**	**P-value**
Number	350	91	
Mean age (SD)	60 (13)	49 (11)	0.00001
Mean size (SD)	19 (14)	25 (18)	0.008
pT			
< 20 mm	216 (62%)	45 (49%)	0.04
≥ 20 mm	134 (38%)	46 (51%)	
Tumor grade			
G1	69 (20%)	7 (8%)	0.02
G2	169 (48%)	41 (45%)	NS
G3	112 (32%)	43 (47%)	0.01
Nodes			
N positive	78 (22%)	26 (29%)	NS
N Negative	272 (78%)	65 (71%)	
Estrogen receptors			
Positive (>1%)	301 (86%)	72 (79%)	NS
Negative	49 (14%)	19 (21%)	
Progesterone receptors			
Positive (>1%)	266 (76%)	58 (64%)	0.02
Negative	84 (24%)	33 (36%)	
Mean Ki-67 (SD)	20 (14)	27 (20)	0.003
Molecular classification			
Luminal A	160 (46%)	21 (23%)	0.001
Luminal B	141 (40%)	51 (56%)	0.02
HER2	17 (5%)	5 (6%)	NS
Triple negative	32 (9%)	14 (15%)	NS

**Table 2:** Clinicopathological characteristics in the premenopausal women

	**European**	**Arabic/Moroccan**	**P-value**
Number	86	56	
Mean age (SD)	43 (4)	42 (15)	NS
Mean size (SD)	22 (14)	24 (17)	NS
pT			
< 20 mm	47 (55%)	30 (54%)	NS
≥ 20 mm	39 (45%)	26 (46%)	
Tumor grade			
G1	12 (14%)	1 (2%)	0.02
G2	42 (49%)	27 (48%)	NS
G3	32 (37%)	28 (50%)	NS
Nodes			
N positive	23 (27%)	18 (32%)	NS
N Negative	63 (73%)	38 (68%)	
Estrogen receptors			
Positive (>1%)	71 (83%)	45 (80%)	NS
Negative	15 (17%)	11 (20%)	
Progesterone receptors			
Positive (>1%)	61 (71%)	37 (66%)	NS
Negative	25 (29%)	19 (34%)	
Mean Ki-67 (SD)	24 (18)	29 (20)	0.04
Molecular classification			
Luminal A	35 (41%)	10 (18%)	0.01
Luminal B	36 (42%)	35 (63%)	0.02
HER2	4 (4%)	3 (5%)	NS
Triple negative	11 (13%)	8 (14%)	NS

## Discussion

The study of possible ethnic differences in breast cancer has mainly occurred in the United States [12]. Recently, new data have been described for Arab populations, but the true prevalence of breast cancer remains uncertain [4,13-17]. For the majority of these studies, which were performed only on indigenous populations, some clinicopathological features are repeatedly observed: the age of cancer presentation is a decade earlier than in European or US patients, the average size of the lesions is often greater than 20 mm, and the grade of tumors is often higher. Interestingly, these data were corroborated in our study of Arab/Moroccan immigrants. Indeed, in our series, the mean age of presentation of breast cancer was 49 years old in Moroccan patients and occurred almost a decade earlier than in European patients (mean age: 60 years old; p = 0.00001) (Table 1). These data are similar to those recently published by Chouchane et al. in a review article, in which the mean age of presentation of breast cancer for Arab patients was 48 years old, compared to 63 years old in European women. These authors also observed that two-thirds of the Arab patients with breast cancer are younger than 50 years old [4]. We found similar results with our Arab immigrant population. Indeed, 61% of the Arab patients with cancer were less than 50 years old, compared to only 24% in the European population (p = 0.0001) (Table 2). However, these results should be interpreted with caution as they may simply reflect that the immigrant population is on average younger than the European population and that the Arab older patients have a different perception of breast cancer and therefore do not participate in the screening program. These challenges and barriers to breast cancer screening in Arab women have been demonstrated in several studies [17-19].

The second observation on Arab patients frequently found in the literature is the presence of larger tumors (mean tumor size greater than 20 mm) and a higher grade (grade 3) at diagnosis [20,21]. We were able to confirm these findings in the general population used in our study. Indeed, over 50% of the Arab patients had tumors larger than 20 mm, compared to 38% of the European patients with a tumor greater or equal to 20 mm (p = 0.04). Furthermore, the average tumor size was 25 mm in the Arab patients, while it was only 19 mm in Europeans (p = 0.008) (Table 1). We also demonstrated that the Arab patients had an increased incidence in grade 3 tumors (p = 0.01) and fewer grade 1 tumors (p = 0.01) than Europeans.

However, the data concerning the tumor size and grade are only statistically significant in the general population, with all ages combined. Indeed, if we restrict our analysis to the premenopausal women under 50 years old, the differences in the tumor size and grade are less obvious (Table 2). This phenomenon is consistent with recent publications that establish a strong link between age at diagnosis and the size and/or grade of the tumor [7,22]. Molecular subtyping of breast cancer gene expression has resulted in a better understanding of breast carcinoma and several distinctive breast carcinoma molecular subtypes have been identified [22,23]. In addition to this gene expression analysis, immunohistochemical surrogates have been used for breast cancer classification with relatively good reproducibility [24]. In the present work, we demonstrated that the luminal subtype B was the most common in an Arab/Moroccan immigrant population. This subtype was found in 56% of the women in the general population but only in 40% of the European women (p = 002) (Table 1). In contrast, the luminal A subtype was significantly more common in the European (46% than in the Arab patients (23%; p = 0.001). Interestingly, these statistically significant differences in the incidence of luminal A and B subtypes between the two populations were also seen in patients under 50 years old (Table 2). However, no differences were observed for the subtypes HER2 and triple negative. Our results corroborate the study performed on a native population in Morocco, where the luminal B subtype was also the most common [6]. Deregulation in both genomic and/or proteomic expression of cytokeratin 8/18 and TFAP2C (a member of the AP-2 family) has been shown to regulate expression of the ER, and the RET proto-oncogene might contribute to the high proportion of the luminal B subtype observed in Arab women. However, this observation should be confirmed in future studies [25].

## Conclusions

Arab immigrant patients with breast carcinoma have different clinicopathological features from those of European women. In particular, the average age at presentation is almost a decade earlier than in European women and appears as a key factor that at least partially explains the other variable modifications, including the lesion size and histological grade. Moreover, independent of age, the immunohistochemical profile of the molecular classes is different, with more luminal B and less luminal A subtypes, suggesting that there are clinicopathological differences, as well as disparities in the expression profiling in these women.

## Abbreviations

ER: Estrogen receptor; PR: Progesterone receptor; HER2: Human epidermal growth factor receptor 2; FISH: Fluorescent *in situ* hybridization; CEP 17: Chromosome 17 centromere; TFAP2C: Transcription factor AP-2 gamma; AP-2: Activating protein 2; RET: Proto-oncogene tyrosine protein kinase receptor.

## Competing interests

The authors declare that they have no competing interests.

## Authors’ contributions

All authors analyzed, interpreted, and approved the final manuscript.
